# Multiscale Region-Based Convolutional Neural Networks for 3D Object Detection with LiDAR Sensors

**DOI:** 10.3390/s26041156

**Published:** 2026-02-11

**Authors:** Wei-Jong Yang, Song-Bo Yao, Jar-Ferr Yang

**Affiliations:** 1 Department of Electrical Engineering, National Kaohsiung Normal University, Kaohsiung 824, Taiwan; wjyang@mail.nknu.edu.tw; 2 Department of Electrical Engineering, National Cheng Kung University, Tainan 701, Taiwan; q36104187@gs.ncku.edu.tw; 3 System-on-Chip Research Center, National Cheng Kung University, Tainan 701, Taiwan

**Keywords:** 3D object detection, region-based convolutional neural networks, voxel-based sparse convolution, lidar point cloud

## Abstract

LiDAR-based 3D object detection is essential for autonomous driving vehicles under poor lighting conditions. With LiDAR data, point cloud technologies have become increasingly important, as LiDAR sensors are largely cost down. However, the sparsity of point cloud poses a challenge for 3D object detection, requiring advancements in sparse convolutional networks. Given that the multiscale feature fusion mechanism can improve object detection performance using rich information across scale features, we added a refinement fusion network with cross-attention modules to existing 3D voxel-based object detection networks. We also employed a realistic strategy to refine existing point cloud data augmentation techniques to enable the trained detection networks to achieve substantially improved results. The experimental results demonstrate the effectiveness of our proposed detection system across three categories on the KITTI dataset. These enhancements address the limitations of current approaches and highlight the superior performance of the proposed system.

## 1. Introduction

The rise of autonomous driving cars and vehicles with advanced driving assistance systems has sparked increasing interest in three-dimensional (3D) object detection in academia and industry. Without using RGB images under poor lighting conditions, LiDAR with deep learning capacity plays a pivotal role. Compared to image methods, LiDAR offers enhanced accuracy in 3D space and robustness against various weather and lighting conditions. Some studies [1,2] have fully utilized LiDAR–image fusion for effective 3D object detection. TransFusion [1], based on effective transformers, is a robust LiDAR–camera 3D detection framework that uses a soft association mechanism, while IS-Fusion [2] is an innovative multimodal fusion framework that jointly captures instance and scene-level contextual information to achieve excellent multimodal 3D object detection. However, detection networks cannot adapt to severe weather conditions or seasonal changes. When image sensors fail, LiDAR–image collaborations also fail to function correctly. Without image data, 3D object detection networks can still precisely acquire objects’ geometric outlines with detailed 3D information. Thus, based on LiDAR data only, deep learning techniques can still be used [3,4,5,6,7,8,9,10,11,12,13,14,15].

**Figure 1:**
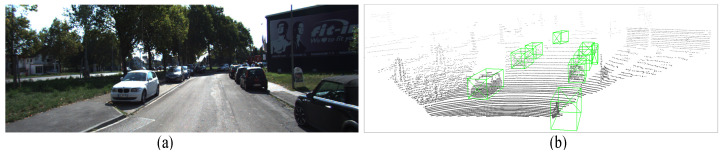
The sampled 3D dataset: (**a**) RGB image; (**b**) point clouds with 3D ground truth labelled.

Figure 1 shows a sample pair of image and LiDAR point cloud data. Generally, 3D object detection networks with LiDAR point clouds can be categorized into point-wise and voxel-wise detection approaches. Point-wise 3D detection utilizes multilayer per-ceptron (MLP) and grouping techniques to extract local features at the point level while preserving certain aspects of the original point cloud information. However, the point-wise approach demands heavy computation inferentially with limited accuracy improvements. Voxel-wise 3D detection leverages the rasterization of point cloud scenes and 3D sparse convolution to extract 3D point cloud information. This approach is steadily gaining prominence as the prevailing approach for point cloud object detection due to the efficiency of convolution operations. Furthermore, with the implementation of suitable refinement frameworks, voxel-wise 3D detection achieves comparable or even higher accuracy than point-wise 3D detection. To accurately predict 3D objects in sparse point cloud scenarios, the use of a precise and efficient 3D object detection network is imperative. Voxelization and voxel feature extraction (VFE) are highly effective for representing sparse point clouds. By discretizing the point cloud scene into a four-dimensional tensor, we can employ a convolutional network on this tensor to ex-tract three-dimensional spatial information. Compared to point-wise operations, the voxel-based approach not only conserves computational resources and inference time but also aligns better with the requirements of self-driving applications.

In this paper, we adopt voxel R-CNN [3] as our primary architecture, as voxel R-CNN effectively enhances feature maps of various scales to achieve better performance. However, the voxel R-CNN lacks a robust mechanism to effectively fuse the distinct scale feature maps. Consequently, we design a two-stage detection network to achieve exceptional accuracy and efficient inference time. We also introduce a novel prediction header to the original voxel-wise 3D detection architecture by enhancing the network to learn from multi-scale objects by complementing feature maps of varying depths and reinforcing the correlation between the region of interest (RoI). Finally, to improve the training process, we combine existing approaches by introducing new data augmentation techniques to further improve the network’s accuracy.

## 2. Related Work

In self-driving systems, 3D object detection based on point cloud data is critical. For instance, military and surveillance applications, such as self-driving drones and un-manned ships, must be able to detect objects without a light source. Likewise, in severe lighting conditions, LiDAR-only 3D object detection is crucial. Over the last years, there has been increased interest in 3D object detection research. There are not only many 3D detectors with distinct architectures but also notable differences in processing point cloud data and extracting point cloud geometric information [3,4,5,6,7,8]. In the following subsections, we review some notable networks used for point cloud object detection and classification.

### 2.1. PointNet and PointNet++

PointNet [4] is based on three key characteristics of point cloud data: (1) they are unordered, (2) there is interaction among points, and (3) there is invariance under transformations. Among these, their unordered nature is most important. PointNet is unordered through symmetric operations, mitigating the potential loss of 3D spatial information by employing multilayer perceptron (MLP) layers. On the other hand, PointNet++ [3] addresses the limitations of PointNet in extracting local features from 3D spatial information by introducing set abstraction (SA) layers. These SA layers employ a farthest point sampling (FPS) and grouping mechanism inspired by the PointNet architecture.

### 2.2. Point-Based 3D Object Detection

Given the good performance of PointNet and PointNet++ in point-wise feature extrac-tion, many networks have adopted these approaches for conducting 3D object detection research on pure point clouds. Building on the original SA layer architecture, 3DSSD [6] introduces a novel approach called F-FPS, which performs FPS operations on the feature coordinate system. Unlike the original SA layer, which uses Cartesian coordinates for FPS (D-FPS), 3DSSD samples key points based on feature distance to select foreground points. Furthermore, 3DSSD introduces a candidate generation (CG) layer designed to predict the offset between each foreground point and the centroid of the object of in-terest. By applying the calculated offset to each foreground point, the CG layer generates predicted candidate points. These candidate points are then fed into the detection head to produce the final regression and classification results.

### 2.3. Voxel-Based 3D Object Detection

Although point-based 3D detection networks handle the sparsity of point cloud data effectively, feature extraction based on individual points requires significant computational resources and time. For real-time applications, point-based 3D detection networks face significant challenges. Recognizing the efficiency of 2D convolution in image detectors, the incorporation of 3D convolution in point clouds has gained momentum. SECOND [7] sequentially feeds point clouds into the 3D and 2D backbones and leverages sparse convolution and sub-manifold sparse convolution [8] techniques to achieve substantial improvements in both accuracy and speed. To convert point clouds into a pixel-like representation, it can be treated as first-step voxelization. To reduce the computation, voxelization based on a predefined voxel size and point cloud ranges segments the entire point cloud scene into several small cubes. Following voxelization, voxel feature extraction (VFE) should be performed to transform multiple point clouds within each voxel into a one-dimensional vector that serves as a feature representation of the voxel. This process generates four-dimensional tensor data that can be used for subsequent 3D convolution operations.

Because the number of non-empty voxels is typically very small compared to the entire scene (usually less than 0.1%), the voxel data are stored in a sparse format, re-cording only the values and indices of the non-empty voxels. SECOND only applies sparse convolution and sub-manifold sparse convolution operations to non-empty voxels, significantly improving feature extraction efficiency. After the features are extracted in 3D space, the feature map is projected onto a bird’s eye view (BEV). Subsequently, the feature map is processed through a 2D backbone network and a region proposal network (RPN) to further extract relevant information and predict the object’s class and 3D bounding box.

### 2.4. Two-Stage 3D Object Detection

Based on single-stage voxel-based detectors and their efficacy in detecting point cloud objects, efforts have been made to extend the concept of two-stage networks from 2D convolutional neural networks (CNNs) to 3D CNNs. For instance, voxel R-CNN incorporates a supplementary refinement network alongside the primary architecture of SECOND. This refinement network comprises two modules: the voxel region of interest (RoI) pooling and a novel prediction head.

The first stage of the network is similar to that of SECOND. However, in the second stage, the network focuses on the RoI features by combining the 3D backbone feature maps with the voxel RoI pooling operation. The process starts by uniformly creating grid points within the RoI. Voxel query is then conducted using these grid points as the centers. The goal is to aggregate non-empty voxels based on their indices. Unlike the ball query used in PointNet++, voxel query uses the Manhattan distance to identify targets within the specified range, rather than a spherical radius. Once the voxel query is complete, the PointNet network generates a dimensional vector that serves as the feature representation for the grid points. This summarizes the process of the voxel RoI pooling module. Once the features of the grid points are obtained, the detection head predicts the refined results.

## 3. Proposed Methods

When the system comes to 3D detection tasks, accurately identifying multiple types of objects within sparse point clouds is crucial. However, due to the inherent limitations of point cloud data in open scenes, the density of point clouds collected by LiDAR for objects of different sizes can vary significantly. This variability poses a challenge when attempting to incorporate mechanisms that effectively fuse the features at different scales.

### 3.1. The Proposed Network Structure

**Figure 2:**
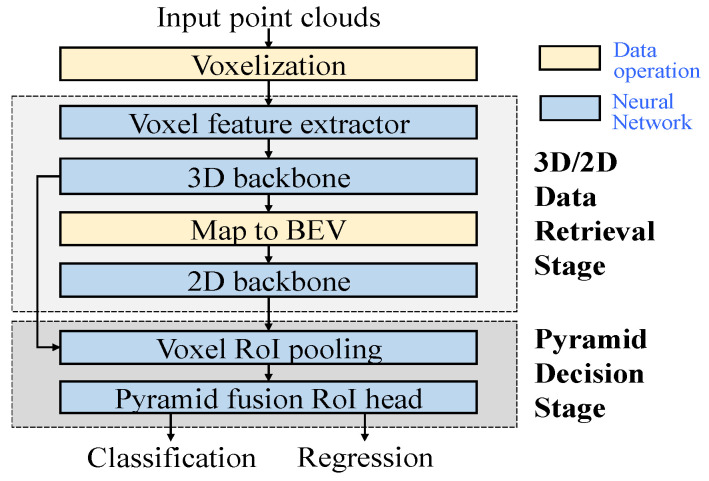
Flowchart of the proposed 3D object detector.

A flowchart illustrating the proposed 3D object detection network is shown in Figure 2. The network takes the point cloud as the input, where each point in the point cloud is represented by its Cartesian coordinates (*x*, *y*, *z*) and reflection intensity *r*. Prior to entering the first stage of the network, the point cloud undergoes voxelization, which divides the *N*-point cloud data into m non-empty voxels. Each voxel can contain up to *n* points, where *m* and *n* represent the maximum number of voxels and the maximum number of points per voxel, respectively. If the number of points exceeds *n*, they should be down-sampled to *n*; if it falls short, the voxel is padded with zeros.

**Figure 3:**
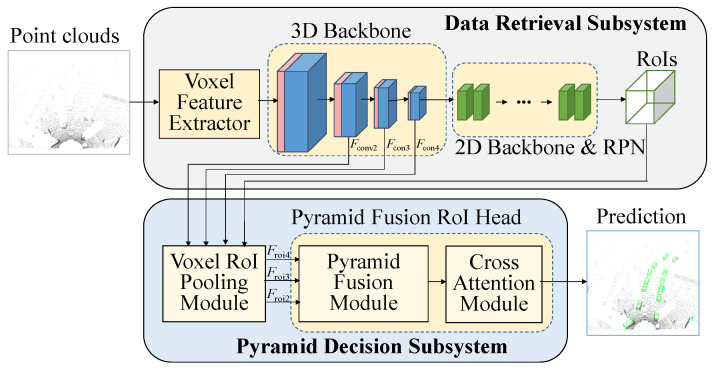
Structure of the proposed 3D object detection network.

Following voxelization, the 3D/2D data retrieval subsystem generates the regions of interest (RoIs), as illustrated in Figure 3. These RoIs contain information on box centroids, sizes, orientations, and classifications. They are then combined with the 3D backbone feature map and fed into the second subsystem of the network, where further refinement of the RoIs takes place for more accurate predictions. The data retrieval subsystem encompasses the voxel feature extractor and 3D backbone and 2D backbone networks. The pyramid decision subsystem includes voxel RoI pooling, pyramid fusion, and cross-attention modules.

### 3.2. Data Retrieval Subsystem

**Figure 4:**
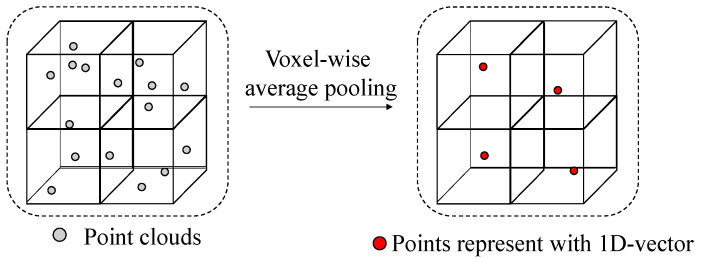
Detailed architecture of the voxel feature extractor (VFE) module.

The data retrieval stage network follows the same architecture as voxel R-CNN [3]. After voxelization, in order to perform 3D convolution, it is necessary to convert the voxel features into one-dimensional vectors. This is achieved by extracting the internal point cloud information using the voxel feature extractor (VFE) module, as illustrated in Figure 4. We employ average pooling for the points within each voxel instead of using PointNet operations [9]. It is not necessary to export an excessive number of channels in the VFE module. Thus, average pooling of the points suffices.

**Figure 5:**
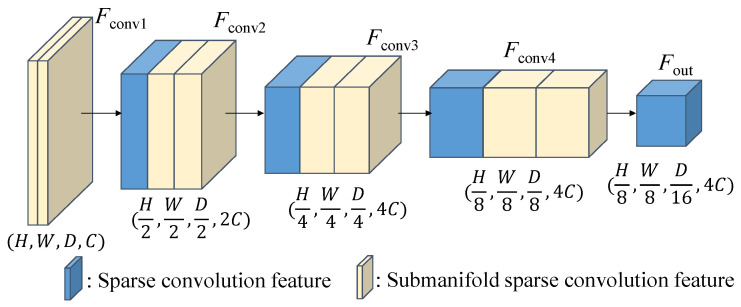
Detailed architecture of the 3D backbone network.

As illustrated in Figure 5, the 3D backbone of our proposed network employs a combination of sparse convolution and sub-manifold sparse convolutions. This approach allows us to handle the sparsity of the data by storing them in a sparse tensor format where only the feature values and indices of non-empty voxels are recorded. Sparse convolution operations are then performed exclusively on these non-empty voxels. This sparse tensor representation serves as a dense feature map with dimensions (*H*, *W*, *D*), resembling a 3D image. For the KITTI 3D dataset, we set the point cloud ranges [*x*_min_, *x*_max_], [*y*_min_, *y*_max_], and [*z*_min_, *z*_max_] as [−40, 40], [0, 70.4], and [−3, 1] meters, respectively, and define the voxel size (*v_x_*, *v_y_*, *v_z_*) to (0.05, 0.05, 0.1) meters. As the dense feature map’s size is determined by dividing the point cloud range by the voxel size, the resulting dimensions of the (*W*, *H*, *D*) feature map in Figure 5 are set to (1600, 1408, 40).

**Figure 6:**
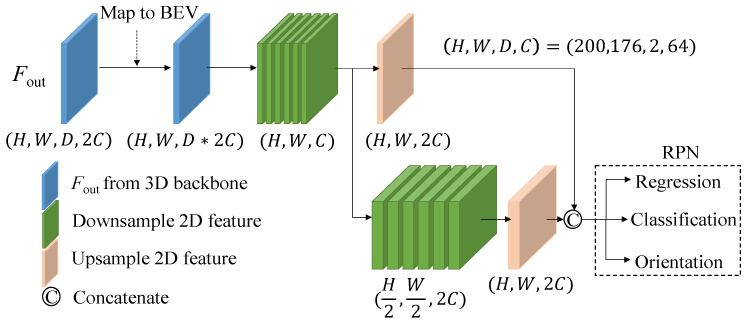
Detailed architecture of the 2D backbone and RPN network.

The 2D backbone and region proposal network (RPN), as depicted in Figure 6, employ the same 2D backbone module utilized in voxel R-CNN [1], although it can be substituted with any other 2D object detection network as desired. The data initially undergoes a conversion from sparse to dense format and is then transformed into a bird’s eye view by reshaping the *z*-axis and channel dimensions to match that of a 2D image. Subsequently, the data is processed by the 2D backbone network to generate RoI proposals, which are used in the subsequent refinement network. It is emphasized that, at this stage, the generated RoIs do not differentiate between foreground and background objects; instead, they preserve the complete anchor-based feature maps. The detailed descriptions of fusion modules (FMs), up-conversion (Upconv) block, and Deep ASSP module will be explained in the following subsections.

### 3.3. Pyramid Decision Subsystem

**Figure 7:**
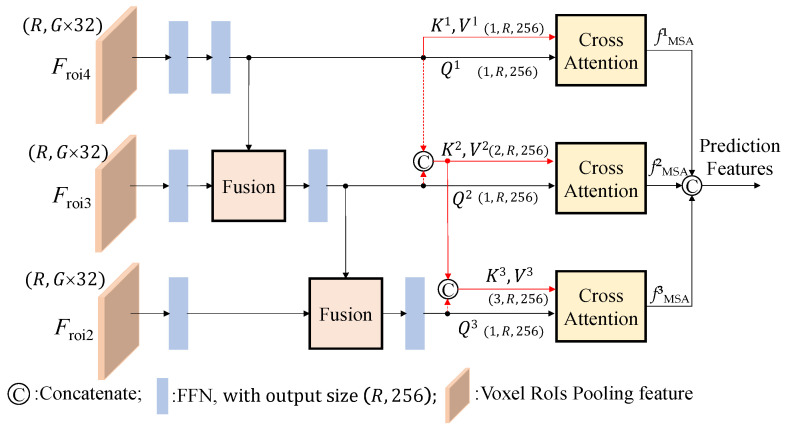
Detailed architecture of the multiscale pyramid fusion RoI head module.

Based on previous work [10,11], we apply cross-attention modules, which lev-erage multi-scale CNN features to enhance 3D object detection. Following the same procedure used in voxel R-CNN [3], the RoIs combine *F*_conv2_, *F*_con3_, and *F*_con4_ features from the 3D backbone, and the voxel RoI pooling operation is performed to generate the multiscale RoI features, *F*_roi2_, *F*_roi3_, and *F*_roi4_. The proposed pyramid fusion RoI head module, which contains fusion modules and cross-attention modules, is used to im-prove the detection results, as illustrated in Figure 7.

**Figure 8:**
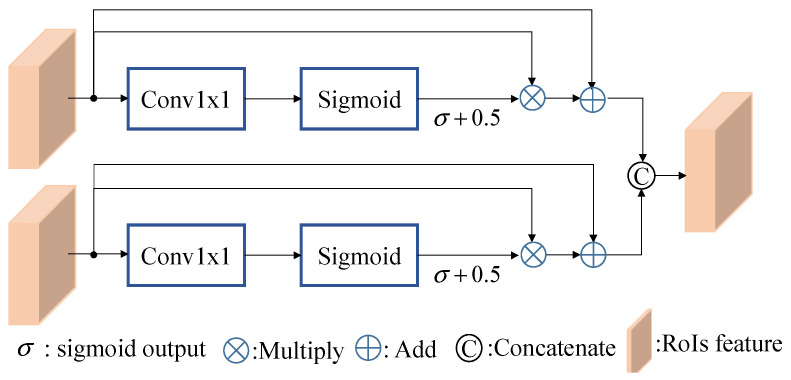
Detailed architecture of the fusion module.

The multiscale pyramid fusion RoI head module shown in Figure 7 is different from that used in the voxel R-CNN, which directly concatenates the multiscale RoI features *F*_roi2_, *F*_roi3_, and *F*_roi4_ and processes them through the FFN network to obtain the final result. We learned that the direct concatenation approach is unable to effectively identify objects across scales and may be unstable when detecting multiple classes. To solve this, we separate the RoI features into 3 levels and enhance the correlation between them using the proposed fusion modules and cross-attention modules, as shown in Figure 7 while the fusion module is shown in Figure 8. This architectural design shares a conceptual similarity with cascade structures, such as the CasA network [12], which employs multiple subnetworks to refine region proposals. However, for practical applications in self-driving systems, this approach exerts a heavy computational burden.

After obtaining the multiscale RoI features with a dimension of (*R*, *G*×32), we pass them through an FFN network to extract the features and adjust the number of channels to (*R*, 256), where *R* represents the number of RoIs. The value of *R* is derived from the output of the data retrieval network and filtered using non-maximum suppression (NMS). In training and testing processes, we set *R* = 512 and *R* = 100, respectively. Additionally, *G* denotes the number of grid points, i.e., *G* = 216, for 6 × 6 × 6 grid points uniformly sampled along the length, width, and height of each box within an RoI.

Next, the relationship between the previous RoI feature and *F*_conv3_ and *F*_conv2_ levels is reinforced using the fusion modules, depicted in Figure 8. Drawing inspiration from the recursive feature pyramid (RFP) module introduced in DetectoRS [13], we observe that the combination of 1 × 1 convolution and sigmoid operations enables the network to learn the weight σ, determining the significance of each feature. However, in the original design, the fusion of the two features in a complementary manner leads to one feature dominating the other, resulting in imbalanced feature representations. To address this limitation, our proposed fusion module allows two RoI features with different scales to learn their respective weight σ. Because σ is an output value in the range of [0, 1], we aim to encompass both weaker and stronger scenarios by adjusting the output range to [0.5, 1.5] by adding a 0.5 offset. Finally, the fusion module concatenates the two features as its output by multiplying the RoI features by their respective weights and incorporating residuals.

**Figure 9:**
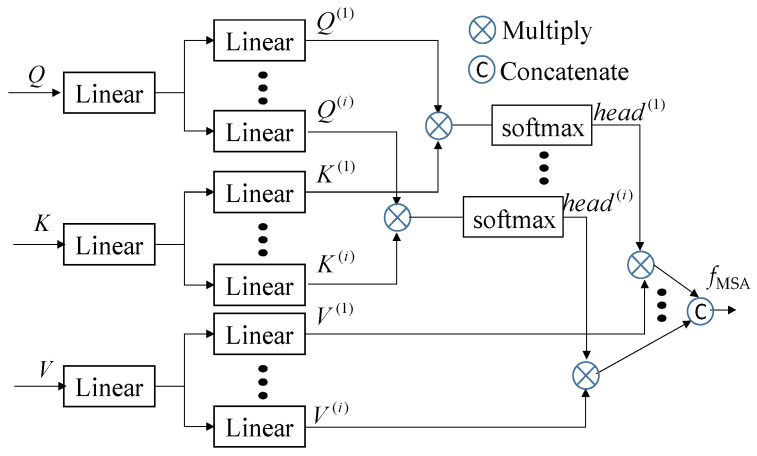
Illustration of cross-attention module with *i* multi-head attention.

To enhance the correlation between different levels, we introduce the cross-attention module at the end of each level [16,17]. This module takes the features of the current level as *Q* inputs and incorporates all of the features from the upper levels, including the current level, as *K* and *V* inputs. The architectural design of this multi-head self-attention process is illustrated in Figure 9. To strengthen the interdependencies among levels, multi-head self-attention is first expressed by *f*_MSA_ = *MSA*(*Q*, *K*, *V*), with *i* set to 4. To enrich the *Q*, *K*, and *V* inputs, we incorporate additional information through position embedding and employ multi-head architectures. Before entering the cross-attention module, the inputs of all levels are transformed into three-dimensional features. As shown in Figure 7, all of the *Q^l^* inputs are unsqueezed from dimension (*R*, 256) to (1, *R*, 256), while the *K^l^* and *V^l^* inputs of the *l*^th^ level features after concatenation of previous level features are unsqueezed to (*l*, *R*, 256). At the first level, the inputs *Q*^1^, *K*^1^, and *V*^1^ are the same, and the first cross-attention module gives the self-attention as *f*^1^_MSA_ = *MSA*(*Q*^1^, *K*^1^, *V*^1^). As shown in Figure 7, the outputs of the *l*^th^ cross-attentions (*f^l^*_MSA_ = *MSA*(*Q^l^*, [*K^l^*; *K^l^*^−1^], [*V^l^*; *V^l^*^−1^]) for *l* = 1, 2, 3 with dimensions matching those of *Q^l^*) are concatenated into a single prediction head to generate the final prediction features.

### 3.4. Polar-Based Data Augmentation

**Figure 10:**
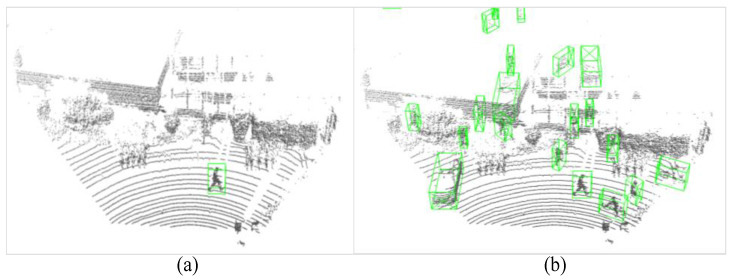
Illustration of original point cloud scenes: (**a**) with ground truth labelled; (**b**) after the gt_sampling data augmentation.

In point cloud scenes, as shown in Figure 10a, the types and quantities of objects present are often unbalanced. For example, in this figure, only one pedestrian is marked in the entire scene, and this imbalance could impact network training. To overcome this, typical 3D object detection networks often employ the gt_sampling data augmentation technique, which aims to increase the number of rare objects in a scene and balance the distribution of different classes, as illustrated in Figure 10b.

**Figure 11:**
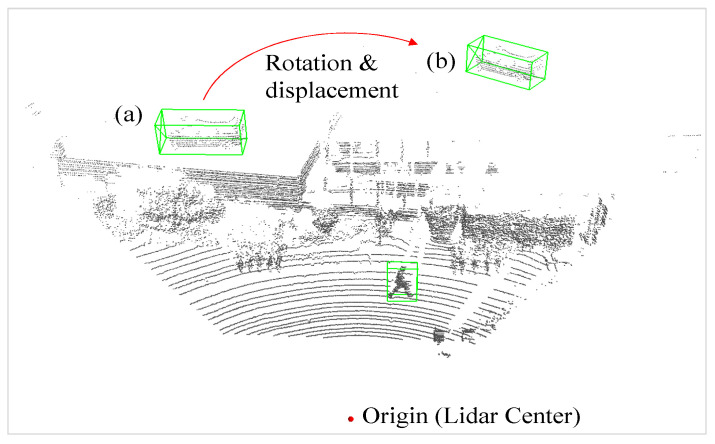
Illustration of improvement for gt_sampling: (**a**) sampled ground truth from other scenes but colliding with building; (**b**) rotated and displaced sampled ground truth for correct data augmentation.

The gt_sampling operation involves randomly sampling ground truth objects from other scenes and subsequently checking for collisions between these sampled objects. If a collision occurs, the corresponding ground truth is discarded. To further enhance the diversity of the augmented data, we suggest an improvement to the original gt_sampling method. As depicted in Figure 11, once a collision is detected between the newly added sampled object and current ground truths, we randomly shift the sampled objects to different locations. Given the nature of LiDAR data, the detected light always emanates from the side of an object facing the origin of the LiDAR source. To avoid a collision, we randomly shift the object by transforming it into a new polar coordinate. We rotate the object based on its radial distance *r* and azimuth angle *θ* to ensure that the point cloud data align with the direction in which the object faces the origin.

**Figure 12:**
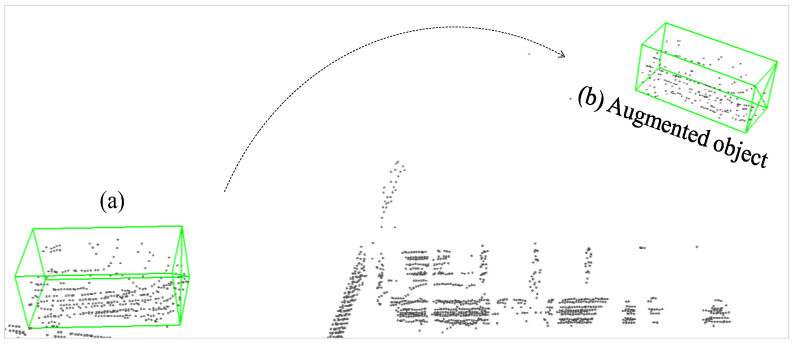
Illustration of distance decay mechanism in our polar-based augmentation: (**a**) original object density; (**b**) the augmented object after points dropout based on radial distance.

Furthermore, the number of objects in point clouds captured by LiDAR tends to decrease when the distance between LiDAR and the object increases. To account for this, we devised a sampling mechanism that enables the object density to decrease with distance. Here, object density refers to the ratio of the number of point clouds belonging to an object to the volume size it occupies. The distance metric employed for this purpose is based on the radial distance *r* from the sensor, as shown in Figure 12. We believe that this decaying sampling design will support the network in gaining more insight into objects with sparse density, ultimately enhancing the network’s performance.

### 3.5. Loss Functions for Training

To train our networks, we employ the same loss functions as those used in the voxel R-CNN. The total training loss is expressed as

(1) $$L_{total}=L_{DR}+L_{PD}$$

where *L_DR_* is loss in the data retrieval stage and *L_PD_* is loss in the pyramid decision stage. We utilize the output of the RPN head as a predictor for the data retrieval stage. 

Loss in the data retrieval stage is defined as

(2) $$L_{DR}=\frac{1}{N_{I}}[\sum_{i}L_{cls}(c_{i},c_{i}^{t})+B({\mathrm{IoU}}_{i}>\theta )\sum_{i}L_{reg}(r_{i},r_{i}^{t})],i\in N_{I}$$

where $N_{I}$ is the number of foreground anchors produced by the RPN head and $c_{i}$, $c_{i}^{t}$, $r_{i}$, and $r_{i}^{t}$ represent the predictions and ground truths of classification and regression, respectively. In (2), $B({\mathrm{IoU}}_{i}>\theta )$ indicates that only the *i*^th^ proposal with intersection over union (IoU) greater than the threshold *θ* will produce the regression loss. The classification loss $L_{cls}$ and the regression loss $L_{reg}$ are binary cross entropy and smooth-L1 loss, respectively. In bounding box regression, the 3D bounding box differs from the 2D bounding box in that it comprises seven-dimensional box parameters. These parameters include the center point (x,y,z), the box size (h,w,l), and the rotation direction *θ* along the *z*-axis.

Similar to *L_DR_*, loss in the pyramid decision stage is defined as

(3) $$L_{PD}=\frac{1}{N_{II}}[\sum_{i}L_{cls}(c_{i},c_{i}^{t})+B({\mathrm{IoU}}_{i}>\sigma )\sum_{i}L_{reg}(r_{i},r_{i}^{t})],i\in N_{II}$$

where *N_II_* is the number of foreground anchors produced by the pyramid fusion RoI head and *B*(IoU*_i_* > σ) indicates that the *i^t^*^h^ RoI with IoU greater than the threshold *σ* will contribute to $L_{reg}$. The IoU thresholds *θ* and *σ* are usually different.

## 4. Experimental Results

**Figure 13:**
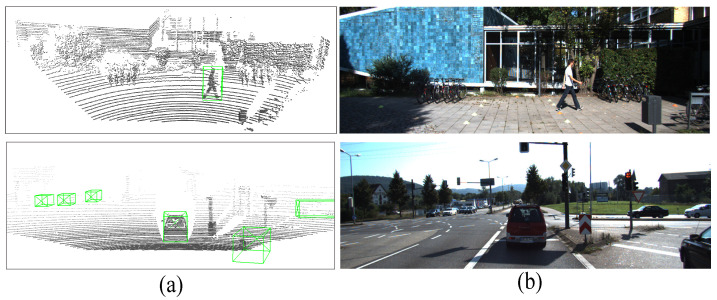
Two examples of KITTI 3D dataset: (**a**) point clouds; (**b**) RGB images.

The proposed 3D object detector is implemented in Python 3.8.16 and PyTorch 1.9.1. For the hardware devices, we used a standard desktop with Intel Core i9-10920X CPU with 3.5GHz and Nvidia Quadro RTX6000 24G GPU with the support of CUDA 11.1. We used the KITTI 3D dataset [18] for both training and evaluation. The dataset consists of 7481 points cloud data samples, which we split into two sets: 3769 samples for the KITTI training set and 3712 samples for the KITTI evaluation set. Furthermore, each piece of point cloud data is accompanied by its corresponding RGB image, providing a visual reference for understanding the scene, as shown in Figure 13a,b. However, the proposed 3D detection networks rely solely on point cloud input and do not incorporate color image information during the training or evaluation tasks.

In the training process, we used the Adam optimizer [19] with β_1_ = 0.9, β_2_ = 0.99. We conducted training for 120 epochs using a batch size of 10 and selected the weight that exhibited the best overall performance across all epochs. The optimal weights were selected from this set of epochs. Additionally, during training, we configured the maximum points per voxel to 5 and the maximum number of voxels to 16,000. During testing, the maximum number of voxels was increased to 40,000. For data augmentation, in addition to our proposed polar-based data augmentation, we applied random rotations, scaling, and *y*-axis flipping operations to the training data.

The KITTI dataset employs average precision (AP) as its evaluation metric, which involves calculating the average of precision values obtained from the precision–recall curve. We used ${\mathrm{AP}|}_{R40}$ instead of ${\mathrm{AP}|}_{R11}$ based on the recommendation of the Mapillary team [20]. The inclusion of zeros in ${\mathrm{AP}|}_{R11}$ can sometimes lead to misleading performance improvements. By using ${\mathrm{AP}|}_{R40}$, we aim to eliminate this potential bias, ensuring fair comparison and better estimation of the area under the precision–recall curve.

### 4.1. Verification of Proposed Method

We primarily used the OpenPCDet toolbox (Team 2020) [21] for implementation, which facilitated the acquisition of pretrained weights for the voxel R-CNN model [3]. Because the voxel R-CNN method has shown better results than most point-wise and voxel-wise methods [3], the proposed method is only compared to the voxel R-CNN approach without loss of generosity.

Table 1 presents the performance comparisons in capabilities of the proposed and voxel R-CNN [3] when the “use_road_plane” technique used in data augmentation is not used in the networks, while Table 2 shows the detection results that adopt the “use_road_plane” technique. Because the objects in different scenes could be on their road planes, which could be uphill or downhill with different slopes, the “use_road_plane” technique suggested for gt_sampling data augmentation vertically adjusts the added ground truths to the road plane of the current scene. Due to the possible geometric non-flat roads and inaccurate road plane estimation, the use_road_plane technique might cause the objects to inexplicably appear below/above the road plane. Under the same conditions, we focus on comparing the detection capabilities of both networks by using identical training batch sizes and epochs. We conducted re-training of the voxel R-CNN model, where the retraining results are marked by the symbol “∇” in Table 1 and Table 2. In Table 1 and Table 2 and all reminding tables, the results in bold indicate the best result in that category. If we leveraged the pretrained weights of voxel R-CNN released in the OpenPCDet toolbox, Table 2 shows the computed results for AP|R40, marked with “∗”. The pretrained weights are only available for a single class, “Car”. We marked the unknown results with “-” for the remaining pedestrian and cyclist classes in Table 2.

With the retraining voxel R-CNN, Table 1 shows that the proposed 3D object detection method is better than the voxel R-CNN method for all car, pedestrian, and cyclist classes. If we adopt the use_road_plane technique in data augmentation, Table 2 shows the performances in AP|R40 metric achieved by the proposed 3D detection system and the voxel R-CNN method. Except for large objects, the “use_road_plane” technique slightly improves detection performance. During the retraining process, we strictly adhered to the configuration and dataset specifications provided in the OpenPCDet toolbox and maintained a batch size of 16, as indicated in the original paper [3]. According to Table 1 and Table 2, the detection precision of the proposed method and the retrained voxel R-CNN cannot improve the detection of large objects using the use_road_plane technique.

### 4.2. Discussion and Ablation Study

To verify the necessity of the proposed designs, in this subsection, we evaluate the performances of various combinations of the proposed modules and submodules through intensive ablation studies. We modified the structures of the networks and retrained them using the same configuration provided in the OpenPCDet toolbox. The “use_road_plane” technique for data augmentation was not used for evaluations. The mean average precision (mAP) was calculated from three difficulty levels (easy, moderate, and hard). The total averaged performance metric mAP, which is denoted as “Avg.”, was computed from the averaging performance across all three object categories.

In Table 3 and Table 4, we compare improvements using different proposed modules. In Table 3, we present the results of retraining the original voxel R-CNN network on our test platform and treat it as the “Original” method. The Original^+PF^ method replaces the original detection head with our proposed pyramid fusion RoI head module, while the Original^+PA^ method adopts the polar-based augmentation method. If the original voxel R-CNN network incorporates both the proposed pyramid fusion RoI head module and the polar-based data augmentation method, it will become the Proposed* method. Table 3 shows that the proposed pyramid fusion RoI head module and the polar-based augmentation method can successfully improve the voxel R-CNN’s detection of cars, pedestrians, and cyclists.

Based on the Proposed* method, we kept the polar-based augmentation method and modified the pyramid fusion RoI head module, which contains fusion submodules and cross-attention submodules. The results in Table 4 show that the Proposed^-F^ method removes the fusion submodule; the Proposed^-C^ method deletes the cross-attention submodule; and Proposed^-CF^ removes both the cross-attention submodule and the fusion submodule from the pyramid fusion RoI head module. The proposed fusion submodule could enhance the networks to better detect pedestrians. The networks with fusion submodules focus on smaller objects, which may compromise the detection of larger objects. On the other hand, for the cross-attention module, the referenced multiple-level features tend to average out its performance across various categories. Given this, we decided to include both submodules, as the Proposed* method can achieve the best overall performance by using correlations among the features at various scales.

Table 5 presents comparisons of the order of fusion in the pyramid fusion RoI head module, where fusion can be performed in the order of “shallow to deep” or “deep to shallow”. To fuse the three RoI features, *F*_roi2_, *F*_roi3_, and *F*_roi4_, we treat *F_r_*_oi4_ as the deep feature, while *F*_roi2_ refers to the shallow one. Figure 7 shows the “deep_to_shallow” architecture of the pyramid fusion RoI head module as it starts at *F*_roi4_ and ends at *F*_roi2_. The shallow_to_deep case starts at *F*_roi2_ and ends at *F*_roi4_. Table 5 shows that the proposed deep_to_shallow fusion module illustrated in Figure 7 performs better than the shallow_to_deep structure.

To further explore the proposed multilevel fusion module, Table 6 shows the performance comparisons using different numbers of fusion levels with RoI features *F*_roi2_, *F*_roi3_, and *F*_roi4_. Based on their importance, levels 1, 2, and 3 should use the RoI features {*F*_roi4_}, {*F*_roi4_, *F*_roi3_}, and {*F*_roi4_, *F*_roi3_, *F*_roi2_}, respectively. The deep features contain global and semantic information, while the shallow features contain local and detailed information. The features in lower levels help the networks detect fine and small objects, which could impact the detection of large objects. Table 6 shows the baseline approach’s performance in detecting cars, which directly concatenates all of the related RoI features suggested in the voxel R-CNN and the proposed pyramid fusion RoI head module. The proposed pyramid fusion RoI head module achieves better performance than the baseline approach. For large objects, such as cars, neither method is improved by including more level features. However, the previous results show that they can both achieve better overall performance with the inclusion of more level features.

To further verify the offset design of the proposed fusion module in Figure 8, Table 7 presents a comparison of the designed fusion module in the pyramid fusion RoI head to evaluate whether the sigmoid output includes an additional offset of 0.5. Table 7 shows that while there is no improvement for the car class, overall performance improves if we include the additional offset 0.5.

## 5. Conclusions

In this paper, we introduce a novel 3D object detection network for LiDAR point clouds. We enhanced the voxel R-CNN method by proposing a pyramid fusion RoI head module, which uses a multiscale feature fusion module to combine RoI features from different levels with cross-attention modules to enhance the correlation among these levels. By incorporating these modules, we have improved the overall detection performance of the 3D object detection network by effectively fusing the features at various scales. During the training phase, we also employ the proposed polar-based data augmentation technique to introduce sampled ground truth objects for training. Specifically, we apply displacement and rotation data augmentation strategies while gradually reducing the density of points for objects of increasing distance. This polar-based data augmentation approach, which is more realistic when adding new objects, enables us to achieve better network performance by enriching the training data and better mimicking real-world scenarios. Compared to the voxel R-CNN method, we demonstrate the effectiveness of the proposed methods through comprehensive and rigorous ablation experiments.

## Figures and Tables

**Table 1:** Performance comparisons of the 3D objection detection evaluated on KITTI val set with AP calculated by 40 recall positions for car, pedestrian and cyclist classes without use_road_plane technique in data augmentation.

Methods	Car 3D *AP_R_*_40_ (%)			Pedestrian 3D *AP_R_*_40_ (%)			Cyclist 3D *AP_R_*_40_ (%)		
	Easy	Mod.	Hard	Easy	Mod.	Hard	Easy	Mod.	Hard
Voxel R-CNN ^∇^	92.33	82.63	80.00	62.53	54.86	48.96	89.93	73.97	68.56
Proposed	**92.67**	**83.26**	**80.50**	**66.35**	**58.02**	**52.05**	**91.40**	**75.48**	**70.86**

^∇^ With retrained weights.

**Table 2:** Performance comparisons of the 3D objection detection evaluated on KITTI val set with AP calculated by 40 recall positions for car, pedestrian and cyclist classes with use_road_plane technique in data augmentation.

Methods	Car 3D *AP_R_*_40_ (%)			Pedestrian 3D *AP_R_*_40_ (%)			Cyclist 3D *AP_R_*_40_ (%)		
	Easy	Mod.	Hard	Easy	Mod.	Hard	Easy	Mod.	Hard
Voxel R-CNN *	**92.15**	85.01	82.48	**-**	-	-	-	-	-
Voxel R-CNN ^∇^	91.52	83.81	81.78	**67.95**	**60.68**	**55.33**	90.52	73.28	68.82
Proposed	92.12	**85.02**	**82.74**	67.77	60.09	54.91	**93.42**	**75.18**	**70.43**

* With pretrained weights provided in OpenPCDet toolbox [15]; ^∇^ With retrained weights.

**Table 3:** Performance comparisons of the proposed 3D detection networks with different combinations of the proposed pyramid fusion RoI head module and polar-based data augmentation method evaluated on KITTI val set.

Methods	Pyramid Fusion RoI Head	Polar-Based Augmentation	Car	Pedestrian	Cyclist
			mAP	mAP	mAP
Original			84.98	55.45	77.48
Original^+PF^	**✓**		85.09	57.69	76.70
Original^+PA^		**✓**	**85.71**	55.47	77.99
Proposed	**✓**	**✓**	85.47	**58.80**	**79.24**

**Table 4:** Performance comparison of pyramid fusion RoI head w/o fusion and cross attention submodules evaluated on KITTI val set.

Methods	Fusion Module	Cross-Attention	Car	Pedestrian	Cyclist	Total
			mAP	mAP	mAP	Avg.
Proposed*	✓	✓	85.47	58.80	79.24	**74.51**
Proposed^−F^		✓	85.22	56.51	**79.76**	73.83
Proposed^−C^	✓		84.83	**59.23**	76.28	73.45
Proposed^−CF^			**85.71**	55.47	77.99	73.06

**Table 5:** Performance comparison of pyramid fusion RoI head with different fusion orders evaluated on KITTI val set.

Fusion Order	Car	Pedestrian	Cyclist	Total
	mAP	mAP	mAP	Avg.
shallow_to_deep	83.73	58.02	78.09	73.28
deep_to_shallow	**85.47**	**58.81**	**79.24**	**74.51**

**Table 6:** Performance comparison of the fusion network with different numbers of levels evaluated on KITTI val set for detection of Car class.

Number of Levels	Baseline	Ours
	mAP	mAP.
1	85.33	**85.81**
2	84.99	**85.48**
3	84.64	**85.47**

**Table 7:** Performance comparison of 0.5 offset for sigmoid function in fusion module evaluated on KITTI val set.

Method	Car	Pedestrian	Cyclist	Total
	mAP	mAP	mAP	Avg.
w/o 0.5 offset	**85.92**	57.27	76.01	73.07
w/ 0.5 offset	85.47	**58.80**	**79.24**	**74.51**

## Data Availability

All test data is from the KITTI Vision Benchmark Suite.
